# How important is the Sphenoseptal cell in identifying the skull base?Recent incidence and demonstration of endoscopic surgical steps

**DOI:** 10.3906/sag-2012-187

**Published:** 2021-08-30

**Authors:** Cem SAKA, Bülent ÖCAL, Emel ÇADALLI TATAR, Gökhan TOPTAŞ, Tuğçe PÜTÜRGELİ ÖZER, Mehmet Hakan KORKMAZ

**Affiliations:** 1 Department of Otorhinolaryngology Head and Neck Surgery, University of Health Sciences, Ankara Dışkapı Training and Research Hospital, Ankara Turkey; 2 Department of Otorhinolaryngology Head and Neck Surgery, Ankara Yıldırım Beyazıt University Faculty of Medicine, Ankara Turkey

**Keywords:** Sphenoseptal cell, sphenoid sinus, skull base, anatomic variation

## Abstract

**Background/aim:**

Because of close relations to important anatomical structures such as cavernous sinus and optic nerve, sphenoid sinus variations must be well trained by the otolaryngologist who has an interest in endoscopic sinus surgery. Newly defined sphenoseptal cell (SSC) is one of those variations that may lead to insufficient endoscopic sinus surgery outcomes if not defined preoperatively with imaging studies. The present study aimed to present the main characteristics of this special type of nasal cell.

**Materials and methods:**

In this study, 610 paranasal sinus CT scans were analyzed and reviewed retrospectively between May 2018 and December 2019. Also, endoscopic findings of SSC that cause a surgical catastrophe in identifying skull base and sella are presented during transnasal transsphenoidal pituitary surgery.

**Results:**

According to its definition and relation to the sphenoid sinus and the skull base, an SSC was seen in 21 scans of 610 patients (3.4%), 11 were women (55%) and 10 were men (45%).

**Conclusion:**

Although an SSC is a rare variation of nasal air cells, preoperative diagnosis of this cell is of paramount importance in some patients during endoscopic transnasal surgery for the identification of skull base.

## 1. Introduction

The endonasal transsphenoidal approach is the most commonly practiced operative route for most pituitary adenomas and other sellar/parasellar masses in an era of minimally invasive surgery. This approach provides a better exposure than the classic microscopic technique concerning the use of angled endoscopes and enabling a wider visual field [1–4]. The surgeon has to be well trained and master the surrounding anatomy to prevent surgical complications. Given the varying degrees of the sphenoid sinus pneumatization, the surgical field can be in close relation to the cavernous sinus, the optic nerve, the internal carotid artery, cranial nerves III to VI, the frontal lobe, and the pituitary gland. Given the special interest in the literature on the Onodi cell, which lies superolateral to the sphenoid sinus and is considered part of the ethmoid complex, several reports have presented its variations and suggested reliable methods to identify them [5,6]. The sphenoseptal cell, which originates from the posterior nasal septum and extends into the sphenoid sinus proper, was first defined in 2012 [7]. We describe here this unique anatomical variant of paranasal sinuses in detail with the new surgical and scanning findings. 

## 2. Materials and methods

A retrospective analysis of paranasal sinus CT scans obtained from adult patients treated in the outpatient clinic of our department from May 2018 to December 2019 was performed. The paranasal sinus CT scans of a total of 610 patients were reviewed and analyzed. Patients who had previously undergone paranasal sinus surgery were excluded. High-resolution axial and coronal CT scanning (slice width at least 1 mm) was conducted for all patients, and sagittal CT images were reconstructed when needed. The CT images were independently screened by 2 otolaryngologists (CS, BÖ), both had more than ten years of surgical experience in rhinology. Any disagreement was resolved by consensus.

### 2.1. The sphenoseptal cell

**Figure 1:**
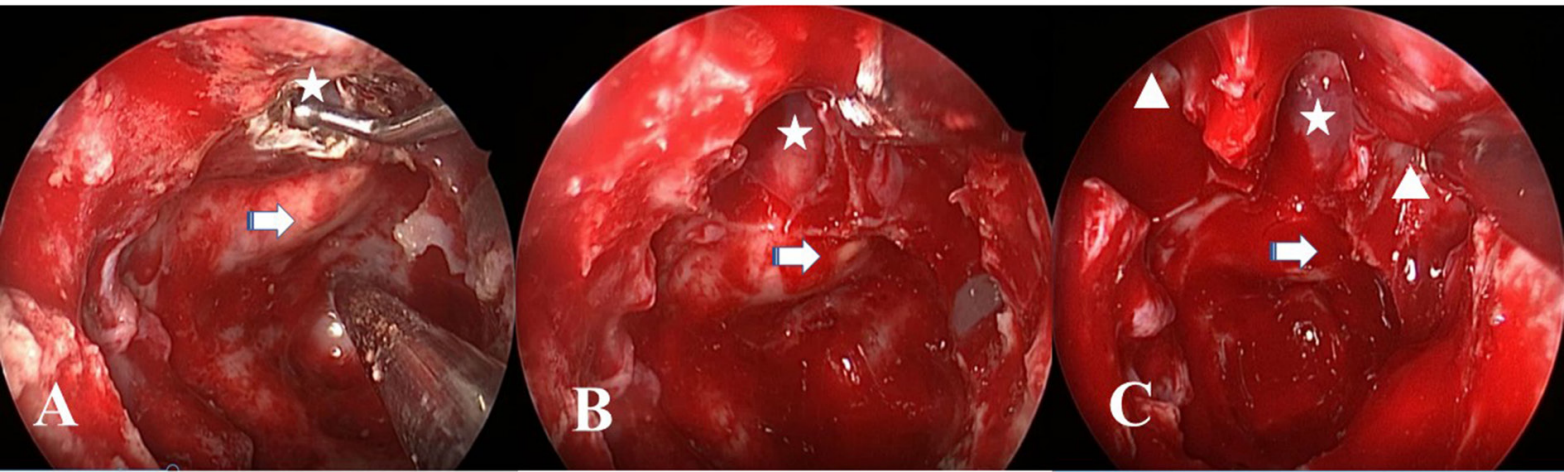
Intraoperative endoscopic views show that a roof of the sphenoid sinus cavity in the midline is dissected (A). The sphenoseptal cell (SSC) (star) is located anterior and superior to the tuberculum sellae (arrow) (B) and between Onodi cells (triangle) on both sides (C).

The sphenoseptal cell (SSC) is identified by evaluating in all three planes (axial, coronal, sagittal). This cell is located superior to the sphenoid sinus in the midline and may stay between the Onodi cells on both sides or can be found alone. Since the SSC is not a part of ethmoidal cells, it is not possible to identify and reach the SSC through the dissection of the ethmoidal cavity. During the endonasal endoscopic approach, after confirming the cell by evaluating images, one has to dissect the roof of the sphenoid sinus in the midline that is immediately anterior to the tuberculum sellae. The presence of the SSC is then confirmed when entering within a cell cavity, which may be separated by bony walls of the Onodi cell on both sides as seen in Figures 1A, 1B and 1C. 

Data regarding patient age are presented as the mean + standard deviation. The descriptive statistics were determined using SPSS (version 23.0; IBM Corp., Armonk, NY, USA).

## 3. Results

**Figure 2:**
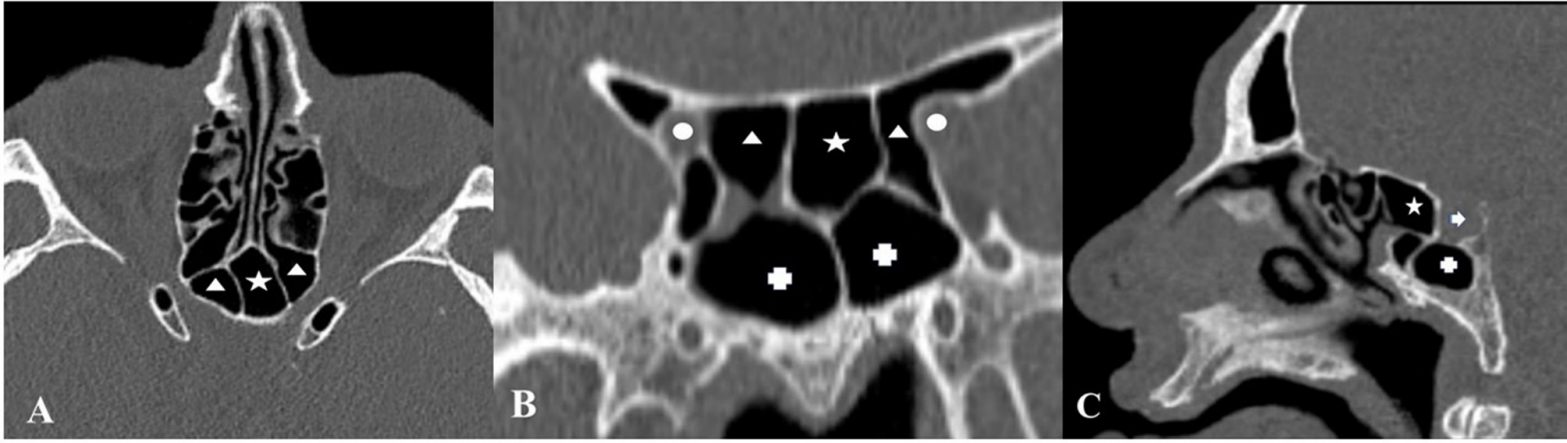
SSC is demonstrated in all three planes. Onodi cells (triangle) attach to the optic nerve (circle) on both sides (Axial (A) and Coronal (B) CT scans). A sphenoid sinus (cross) lying inferior to an SSC (Sagittal (C) and Coronal scans) is shown. A Coronal-sagittal projection of the SSC (Arrow: pituitary gland).

**Figure 3:**
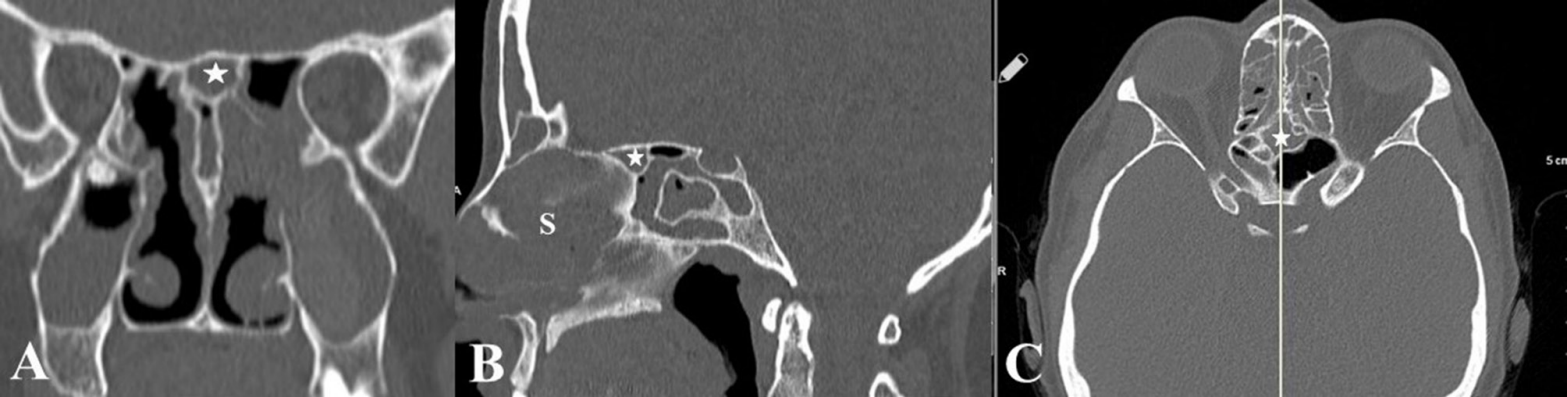
SSC is shown at the skull base (star) on coronal (A), sagittal (B) and, axial images C). This small SSC situates anterior to the Onodi cell and may be easily missed out during an endonasal endoscopic approach (S:nasal septum).

**Figure 4:**
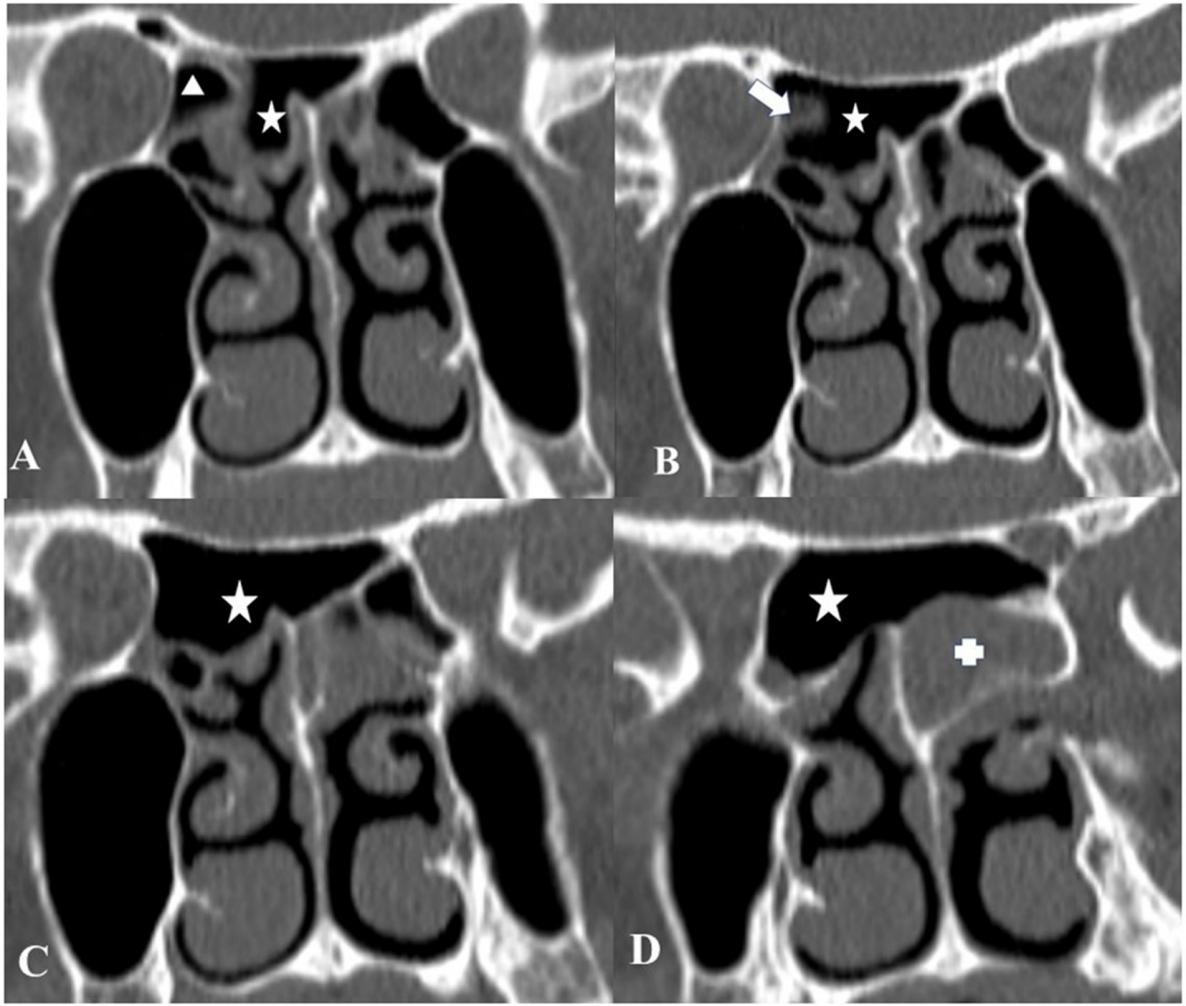
Coronal CT scans of the paranasal sinuses show an SSC (star) on consecutive sections. A right posterior ethmoid cell (triangle) is seen medial to the orbit (A). A posterior wall of this cell (arrow) is anterior to the optic canal (B). Note that an SSC (star) is extending beyond this posterior ethmoidal cell laterally (C) and attaches to optic nerves bilaterally (D).

The paranasal sinus CT images of 610 patients ranging in age from 18 to 76 years (mean, 36.96 + 12,69 years) were studied. The SSC was present in 21 (3.4%) patients. There was no sex difference in prevalence (11 women (55 %), 10 men (45%)). Of these, 5 (23.8%) had also Onodi cells accompanying the SSC as shown in Figures 2A, 2B and 2C. There were cases of the SSC that may occupy a small space in the skull base as presented in Figures 3A, 3B and 3C. Those cases may easily be misdiagnosed and may contribute to insufficient surgeries. There were 16 patients (76.2%) with the SSCs stemming from the posterior of the nasal septum, which extended to the sphenoid sinus superiorly, and attached to optic nerves on both sides as demonstrated in Figures 4A, 4B 4C and 4D.

## 4. Discussion

Surgeons need to be aware of the details of the sphenoid sinus anatomy when performing ESS as well as endoscopic approaches to the sella, parasellar and paraclival structures. The extent of pneumatization of the sphenoid sinus and adjacent structures varies considerably case by case, which can make surgery more difficult. Therefore, success in recognizing certain anatomical variants is extremely important to prevent avoidable complications. The sphenoseptal cell described herein is one of those variants that is solely recognized if a surgeon looks carefully at all three plans of CT preoperatively and rules over the three-dimensional anatomy of paranasal sinuses and related structures. 

Since the most posterior wall of the ethmoid sinus is the anterior wall of the sphenoid sinus, sphenoethmoidal cell variants have gained special interest regarding endoscopic approaches to the sphenoid sinus in the last years. The Onodi cell is the most well-known variant of sphenoethmoidal cells. This cell was originally defined by Adolf Onodi as the posterior-most ethmoid cell that progresses to the lesser wing of the sphenoid. Although the Onodi cell mostly presents a pneumatization in a superior and lateral direction, there are numerous variations of this cell reported [5,8,9].

The anatomical characteristics of sphenoethmoidal cells have been classified into different types depending upon posterior ethmoidal extensions to the sphenoid bone. Wada et al. have focused on the anterior wall of the sphenoid sinus and the optic nerve, skull base, and pituitary gland based primarily on the sagittal CT evaluation findings [8]. In this classification, lateral and medial attachment sites of the anterior wall of the sphenoid sinus present four different types. While the optic nerve stays within the sphenoid sinus cavity in cases of the skull-base type, the nerve runs through the Onodi cell in the other three types. On the other hand, in 16% of the cases, anterior attachment of the sphenoid sinus in the midline clings to the pituitary gland or inferior to the gland. 

The term of the central Onodi cell defined by Cheerla et al. refers to a posterior ethmoid cell extending into the infrasellar region, lying superior and midline to the sphenoid sinus and attaching to the optic canal on both sides [10]. This definition seems to correspond to the fourth type of ethmoid cell defined by Wada et al. Another variation of the posterior ethmoid cell is the inferolateral sphenoethmoid cell, which extends inferolateral to the sphenoid sinus and may cling to the maxillary nerve [5]. All defined cells mentioned here are particularly variants of sphenoethmoidal cells.

SSC has first described by Saka et al. in 2012. This cell is stemming from the nasal septum extending to the sphenoid sinus and push the anterior wall of the sinus backward in the midline. In the case of having SSC, like either type third or fourth cells defined by Wada et al., the sphenoid sinus is lying inferior to SSC; however, this cell must be definitively separated with bony wall/s from the Onodi cell if it exists. One considerable significant clinical scenario is that in the case of SSC not being identified preoperatively by imaging studies because SSC may interfere with exposure of the edge of the sellar floor, one assumes mistakenly the roof of the sphenoid sinus in the midline is the skull base after dissecting the Onodi cells on both sides and getting sphenoidotomy. Instead, the SSC should be entered through the superomedial roof of the sphenoid sinüs, and endoscopic anatomy should be confirmed with the CT images. 

Since the previous study that was first to describe the SSC in 2012 has been carried out in a retrospective manner, the authors had not been able to present intraoperative endoscopic views of this cell. This article includes endoscopic views during a transnasal sellar approach to a pituitary adenoma to confirm the SSC and to show the relationship between this cell and adjacent structures. The incidence of the SSC was found to be higher (three-fold) in comparison with the previous study [7]. This is possible because the previous study sections were evaluated solely on the coronal plane. In the present study, the sections were evaluated in all three plans that conceivably lead to the detection of a higher number of SSCs. 

We can mention as a limitation that the 3D reconstruction of CT imaging would help to better define the imaging criteria of SSC and its presence on preoperative scans.

In conclusion, this article provided radiographic and endoscopic imaging of the SSC. The prevalence of SSC found in the present study was similar to the one previously reported by authors. Preoperative CT scans should be carefully reviewed regarding the superior and lateral boundaries of the sphenoid sinus before surgery to prevent potential complications and to maximize the efficacy of the endoscopic endonasal transsphenoidal sellar and parasellar approaches. 

## Informed consent

University of Health Sciences, Ankara Dışkapı Training and Research Hospital Review Board: 30.11.2020 – 99/18
